# Mapping Climate Change Vulnerabilities to Infectious Diseases in Europe

**DOI:** 10.1289/ehp.1103805

**Published:** 2011-11-23

**Authors:** Jan C. Semenza, Jonathan E. Suk, Virginia Estevez, Kristie L. Ebi, Elisabet Lindgren

**Affiliations:** 1 Office of the Chief Scientist, European Centre for Disease Prevention and Control, Stockholm, Sweden; 2 Department of Medicine, Stanford University, Stanford, California, USA; 3 Institute of Environmental Medicine, Karolinska Institutet, Stockholm, Sweden

**Keywords:** adaptation, climate change, infectious diseases, surveillance, vulnerability

## Abstract

Background: The incidence, outbreak frequency, and distribution of many infectious diseases are generally expected to change as a consequence of climate change, yet there is limited regional information available to guide decision making.

Objective: We surveyed government officials designated as Competent Bodies for Scientific Advice concerning infectious diseases to examine the degree to which they are concerned about potential effects of climate change on infectious diseases, as well as their perceptions of institutional capacities in their respective countries.

Methods: In 2007 and 2009/2010, national infectious disease experts from 30 European Economic Area countries were surveyed about recent and projected infectious disease patterns in relation to climate change in their countries and the national capacity to cope with them.

Results: A large majority of respondents agreed that climate change would affect vector-borne (86% of country representatives), food-borne (70%), water-borne (68%), and rodent-borne (68%) diseases in their countries. In addition, most indicated that institutional improvements are needed for ongoing surveillance programs (83%), collaboration with the veterinary sector (69%), management of animal disease outbreaks (66%), national monitoring and control of climate-sensitive infectious diseases (64%), health services during an infectious disease outbreak (61%), and diagnostic support during an epidemic (54%).

Conclusions: Expert responses were generally consistent with the peer-reviewed literature regarding the relationship between climate change and vector- and water-borne diseases, but were less so for food-borne diseases. Shortcomings in institutional capacity to manage climate change vulnerability, identified in this assessment, should be addressed in impact, vulnerability, and adaptation assessments.

Europe will experience differential impacts from climate change (Intergovernmental Panel on Climate Change 2007). Differences in geographic, ecological, demographic, and socioeconomic conditions affect the region’s differences in vulnerability to changing environmental and climatic conditions. Projections of annual average temperature and mean precipitation predict significant changes overall, with disproportionally warmer winters in the north and warmer summers in the south (Giorgi et al. 2004). Ambient temperature and precipitation patterns influence food- and water-borne diseases through effects on environmental exposure pathways (Semenza et al. 2011a, 2011b). In addition, changes in seasonal precipitation and temperature influence vector-borne diseases through *a*) effects on vector survival, reproduction rates, habitat suitability, distribution, and abundance; *b*) the intensity and temporal pattern of vector activity (particularly biting rates); and *c*) rates of pathogen development, survival, and reproduction within vectors (Semenza and Menne 2009). Thus, projected climate changes may shift the distributional ranges of vector-borne diseases.

There are, however, significant uncertainties in climate change projections, particularly with regard to changes in weather patterns over time and consequences on smaller-scale biogeographic regions. Moreover, complex transmission pathways interact with climatic and environmental factors and are thus often insufficiently understood (McMichael et al. 2006; Patz et al. 2005). It is unlikely that the effect of climate change on a specific pathogen will be idiosyncratic; rather, a multitude of effects are likely to occur because pathogen dispersion, transport, fate, and environmental exposure pathways can all be altered by local climate and weather conditions (Boxall et al. 2009). Although infectious disease outbreaks have been linked to individual weather events, there have been few attempts to detect and attribute temporal trends in infectious diseases to climate change (Semenza et al. 2011b). Many studies have projected future levels of disease spread in response to climate change, but there are currently no means for verifying the accuracy of these models.

Capturing local expert opinion has proven valuable when information is uncertain. Expert assessment and judgment can inform regulators and guide the policy decision-making process (Habegger 2010; Riedy 2009) by identifying climate-related diseases of current or future public health concern as a function of specific vulnerabilities and adaptive capacities (Alberini et al. 2006; Anderegg et al. 2010; King et al. 2006). Therefore, we surveyed national infectious disease experts responsible for climate change activities in their country to evaluate potential impacts of climate change on infectious diseases in Europe and capture information on national assessment plans, the extent to which infectious diseases are covered by those plans, and institutional capacities for managing climate change vulnerabilities.

## Methods

The geographic scope of the survey was defined as the European Economic Area (EEA), which includes all 27 member states of the European Union (EU) plus Norway, Iceland, and Lichtenstein. Governments of these countries designate institutions or scientific bodies to serve as official sources of independent scientific and technical advice and/or capacity for the prevention and control of infectious diseases for the European Centre for Disease Prevention and Control (ECDC). In 2007 and 2009/2010, questionnaires were administered to officials representing these Competent Bodies for Scientific Advice in each member state (ECDC 2011), including representatives from governmental health protection agencies (52%), ministries of health (24%), and governmental infectious disease surveillance centers (24%). Several of the representatives also provided an academic affiliation (14%). A different set of experts was queried in each survey.

The questionnaire asked respondents to indicate which infectious diseases (from lists of 18 and 29 specific diseases in 2007 and 2009/2010, respectively) or groups of infectious diseases (food-borne, water-borne, vector-borne, rodent-borne, parasitic, viral, or other) would most likely be affected by climate change in their respective countries, according to a five-item Likert scale (agree strongly, agree somewhat, neither agree nor disagree, disagree somewhat, disagree strongly). In addition, respondents were asked about numbers of outbreaks within the previous 10 years and whether they believed that some of these outbreaks were attributable to climate change. For endemic insect-, tick-, and rodent-borne diseases, experts were also asked to indicate whether they had observed changes in seasonality and geographic, altitudinal, or latitudinal distributions. The survey instrument also included questions about planning, preparedness, and surveillance.

Likert scale responses were summarized as positive (agree strongly or agree somewhat that climate change will affect disease), negative (disagree strongly or disagree somewhat that climate change will affect disease), or neither agree nor disagree. To assess the reliability of expert responses, two successive interviews with different individuals were conducted over a span of 3 years. Country-level data were pooled to compute summary statistics for the 2007 and 2009/2010 surveys, and a two-sample test of equality of proportions was applied to compare the proportions of respondents with positive and negative responses between the two survey rounds. We used a less stringent level of significance (α = 0.1) to capture minor changes between the two survey rounds. Accuracy of the responses was assessed with data from the peer-reviewed literature and submissions to the United Nations Framework Convention on Climate Change (UNFCCC 2010). Analysis and presentation of the data were performed with ESRI software ArcGIS, version 10.0 (Environmental Systems Research Institute Inc., Redlands, CA, USA).

## Results

Government officials from each of the 30 EEA countries completed a survey questionnaire in 2007, and officials from 29 of 30 countries completed questionnaires in 2009/2010 (nonresponse, Lichtenstein). Different national infectious disease experts were interviewed for the two survey rounds, with an 89% concordance in their assessments; 18 infectious diseases were evaluated in both rounds and for only 2 (chikungunya and dengue fever) did the proportion of countries reporting an impact change significantly (*p* < 0.1).

The majority of country representatives indicated that they believed climate change would have an impact on all major categories of infectious diseases (vector-, water-, food-, and rodent-borne) (Table 1). Diseases with a low or nonexistent disease burden in Europe (e.g., plague, yellow fever, cholera) were ranked rather low (ECDC 2010a). Here we discuss the results by infectious disease category, planning and preparedness, and surveillance activities. For simplicity, summary estimates are reported for the 2009/2010 survey unless otherwise noted.

**Table 1:** Infectious diseases likely to be affected by climate change based on survey responses by infectious disease experts representing EEA countries in 2007 and 2009/2010.

2007 responses			2009/2010 responses			*p*-Value*a*
Disease category	Total (*n*)	Positive [*n* (%)]	Total (*n*)	Positive [*n* (%)]		
Vector-borne diseases		29		25 (86)		29		25 (86)		1
Borreliosis (Lyme disease)		30		25 (83)		28		22 (79)		0.91
Crimean-Congo hemorrhagic fever		27		10 (37)		27		8 (30)		0.56
Chikungunya fever		29		5 (17)		29		15 (52)		0.048
Dengue fever		29		5 (17)		29		11 (38)		0.07
Human granulocytic anaplasmosis		—				24		7 (29)		—
Leishmaniasis		—				27		15 (59)		—
Malaria		29		12 (41)		28		8 (29)		0.33
Q fever		29		12 (41)		26		11 (42)		0.93
Rift Valley fever		29		2 (7)		26		4 (15)		0.32
Tick-borne encephalitis (TBE)		29		21 (72)		29		19 (63)		0.46
Tularemia		29		9 (31)		23		12 (52)		0.1
Viral hemorrhagic fevers		29		5 (17)		25		3 (12)		0.58
West Nile fever		28		15 (54)		27		19 (70)		0.20
Yellow fever		29		2 (7)		27		6 (22)		0.1
Rodent-borne diseases		29		17 (59)		25		17 (68)		0.29
Hantavirus infections		29		18 (62)		27		15 (56)		0.63
Plague		29		1 (3)		25		1 (4)		0.83
Food-borne diseases		29		23 (79)		27		19 (70)		0.42
Water-borne diseases		29		24 (83)		28		19 (68)		0.18
Campylobacteriosis		—				26		14 (54)		—
Cholera		27		4 (15)		23		3 (13)		0.82
Cryptosporidiosis		—				25		10 (40)		—
Enterovirus infections		—				24		12 (50)		—
Giardiasis		—				25		8 (32)		—
Leptospirosis		29		16 (55)		27		15 (56)		0.91
*Naegleria fowleri* infections		—				22		6 (27)		—
Norovirus infections		—				24		6 (25)		—
Rotavirus infections		—				26		7 (27)		—
Salmonellosis		—				27		16 (60)		—
*Vibrio* species		—				24		9 (38)		—
Respiratory diseases		—				—				—
Legionellosis		28		19 (68)		27		16 (59)		0.47
Severe acute respiratory syndrome		29		2 (7)		21		1 (5)		0.74
—, no data for 2007; the list of pathogens was expanded in 2009/2010. There is some overlap between disease categories (not mutually exclusive). Data are based on the following survey question: “Future infectious disease risk in a changing climate; which infectious diseases do you think climate change will most affect in your country?” Responses were recorded on a Likert scale with five response options: agree strongly; agree somewhat; neither agree nor disagree; disagree somewhat; disagree strongly. The first two responses (agree strongly and agree somewhat) were grouped to reflect a positive response. **a***p*‑Values for 2007 compared with 2009/2010.										

*Vector-borne diseases.* Individual vector-borne diseases judged by national experts to be likely affected by climate change in the future included Lyme borreliosis (79%), West Nile fever (70%), and tick-borne encephalitis (TBE; 63%) (Table 1). About one-fourth of respondents also attributed outbreaks or increases in the incidence of these diseases during the last decade to climate change (30%, 25%, and 22% for Lyme borreliosis, West Nile fever, and TBE, respectively). Other vector-borne disease outbreaks (e.g., leishmaniasis, hantavirus infections; data not shown) tend to occur in low numbers or infrequently, which makes attribution to climate change difficult and limits interpretation.

**Figure 1:**
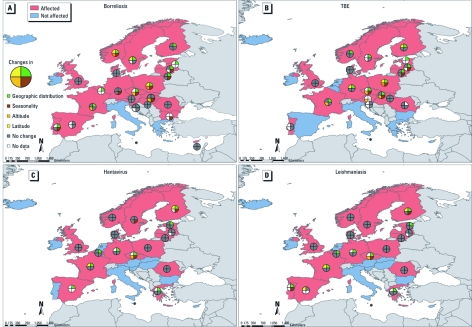
Responses from national infectious disease experts from EEA countries, 2009–2010, as to whether specific vector-borne diseases would be affected or not affected by climate change. (*A*) Borreliosis (Lyme disease). (*B*) TBE. (*C*) Hantavirus infections. (*D*) Leishmaniasis. Data are based on the following survey question on future infectious disease risks in a changing climate: “Which infectious diseases do you think climate change will most affect in your country?”

**Figure 2:**
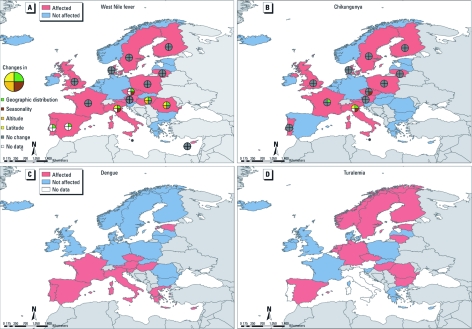
Responses from national infectious disease experts from EEA countries, 2009–2010, as to whether specific vector-borne diseases would be affected or not affected by climate change, 2009/2010. (*A*) West Nile fever. (*B*) Chikungunya fever. (*C*) Dengue. (*D*) Tularemia. No data on changes in the geographic range were collected for nonendemic diseases (dengue and tularemia).

The data for 2009/2010 were mapped by pathogen and country. Potential effects of future climate change on Lyme borreliosis were of concern to respondents from almost all countries except for officials from three Mediterranean countries (Italy, Malta, and Greece) and two Atlantic countries (Ireland and Iceland) (Figure 1A). Respondents in northern and central Europe thought climate change is likely to affect TBE, and respondents in southern Europe expected effects on West Nile fever (Figures 1B and 2A). A larger number of respondents in 2009/2010 than in 2007 felt that climate change would affect chikungunya (52% vs. 17%, *p* = 0.048) and dengue fever (38% vs. 17%, *p* = 0.07) in their countries (Table 1, Figure 2B,C). Experts also reported an increase in the geographic distribution and seasonality of several insect-, tick-, and rodent-borne diseases in their countries in the previous decade (Table 2).

**Table 2:** General changes in insect-, tick-, or rodent-borne diseases in European countries over the last decade, according to infectious disease experts, 2009/2010.*^a^*

Disease	Geographic distribution							Seasonality							Altitude							Latitude						
	Total	+	No change	–	Total	+	No change	–	Total	+	No change	–	Total	+	No change	–
Borreliosis (Lyme disease)		22		11/22		11/22		0		21		9/21		12/21		0		16		5/16		11/16		0		17		3/17		14/17		0
Chikungunya fever		12		2/12		10/12		0		12		2/12		10/12		0		11		0		11/11		0		12		1/12		11/12		0
Hantavirus infections		17		8/17		9/17		0		16		3/16		13/16		0		14		1/14		13/14		0		12		2/12		10/12		0
Leishmaniasis		18		8/18		10/18		1/18		17		3/17		14/17		0		15		1/15		14/15		0		14		3/14		11/14		0
TBE		17		11/17		6/17		0		17		5/17		12/17		0		14		5/14		9/14		0		13		3/13		10/13		0
West Nile fever		16		6/16		10/16		0		14		2/14		12/14		0		12		2/12		10/12		0		13		3/13		10/13		0
Abbreviations: +, range expansion; –, range contraction. Values shown are number of countries reporting change or no change/total number of respondents for each survey item. Data are based on the following survey question: “Have you observed general changes of insect‑, tick‑, or rodent-borne disease in your country over the last 10 years?” Responses were recorded on a scale with five response options: strong increase; increase; no change; decrease; strong decrease. The first two responses (strong increase and increase) were grouped to reflect a positive response and the last two responses (decrease and strong decrease) were grouped to reflect a negative response. Both range expansion and contraction were documented for leishmaniasis in Portugal. Data were not available for 2007.																																

Countries of the northeast reported expansion in the geographic distribution of tick-borne diseases (Lyme borreliosis and TBE) (Figure 1A,B), whereas countries of the south reported expansion in the distribution of mosquito-borne diseases (e.g., West Nile fever; Figure 2A). Both local range contraction and range expansion were reported in the case of leishmaniasis in Portugal (Table 2).

**Figure 3:**
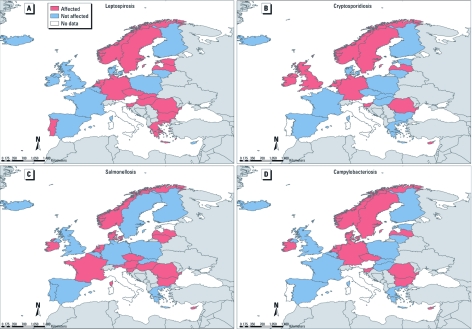
Responses from national infectious disease experts from EEA countries, 2009–2010, as to whether specific food- and water-borne diseases would be affected or not affected by climate change. (*A*) Leptospirosis. (*B*) Cryptosporidiosis. (*C*) Salmonellosis. (*D*) Campylobacteriosis. Data are based on the following survey question on future infectious disease risks in a changing climate: “Which infectious diseases do you think climate change will most affect in your country?”

*Food- and water-borne diseases.* A large proportion of the experts indicated that they believed that water-borne diseases would be affected by climate change in the future (Table 1). Leptospirosis (56%) and cryptosporidiosis (40%) were cited by the largest number of respondents (Table 1, Figure 3A,B). However, respondents from only 3 countries (Finland, Romania, and Sweden) reported increases of water-borne diseases over the last decade (data not shown). Drinking water supplies in Europe can be regarded as a potential source of vulnerability to climate change; for example, the extent of the vulnerability may be determined by whether access is via public (e.g., municipal) or private water systems. Respondents indicated that the proportion of their population using private drinking water sources were 1–10% in 12 countries, 11–20% in 2 countries, 21–40% in 2 countries, and > 40% in 1 country. Responses were missing for 9 countries. In all 5 countries that had > 11% of private drinking water sources, experts considered the country to be at risk of water-borne outbreaks from climate change.

Approximately three-fourths of country experts believed that food-borne infections will be affected by climate change (Table 1). During the most recent survey, the majority of country experts listed salmonellosis (60%), campylobacteriosis (54%), and enterovirus infections (50%) as likely to be affected by climate change in the future (Figure 3C,D). Nearly one-third (9 of 28) of country experts reported an observable increase of food-borne diseases over the last decade, but few attributed the food-borne disease outbreaks to climate change (data not shown).

**Figure 4:**
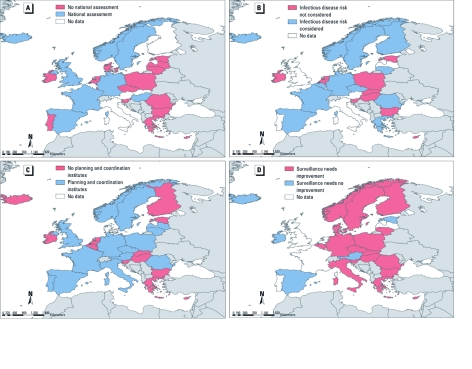
Responses from national infectious disease experts, 2009/2010, as to whether their countries had (*A*) performed national assessments of health impacts from climate change; (*B*) considered infectious disease health risk as a result of climate change; and (*C*) developed local planning and coordination institutes to monitor and control climate-sensitive infectious diseases. (*D*) They were also asked to rate the effectiveness of their country’s surveillance and control programs for vector‑, water‑, and food-borne diseases.

**Figure 5:**
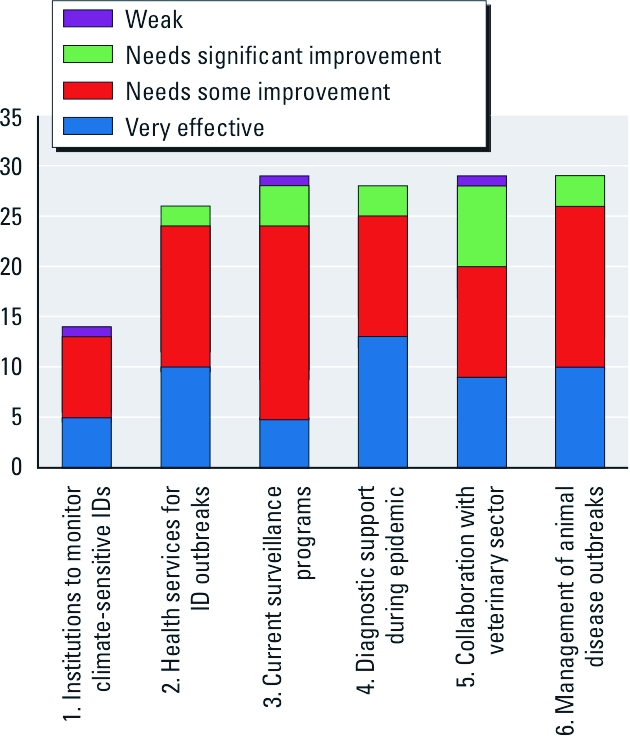
Responses from national infectious disease (ID) experts, 2009/2010, as to the effectiveness of institutions, health services, and surveillance programs for vector-, water-, and food-borne diseases by EEA countries. The *y*-axis represents the EEA countries, which included 27 EU member states plus Norway and Iceland, with the exception of Lichtenstein. Incomplete data are due to missing information. Data are based on the following survey questions: 1. Are there regional or local planning and coordination institutions to monitor and control climate-sensitive IDs? 2. Are regional or local health services able to provide essential health services during an ID outbreak? 3. How effective are current surveillance and control programs for vector-, water-, and food-borne diseases? 4. How effective is their capacity to provide routine and diagnostic support in case of an epidemic? 5. How effective is your collaboration with the veterinary sector with regard to both surveillance and responses to an outbreak? 6. How is an animal disease outbreak, with human health implications, managed in your country?

*Planning and preparedness.* Only 9 of 27 respondents indicated that their countries had completed a national assessment specifically focused on the potential health impacts of climate change (Table 3, Figure 4A). Coverage of infectious diseases by the assessment was reported to be extensive, adequate, or minimal by 4, 3, and 2 respondents, respectively. However, in 14 of 25 countries where adaptation initiatives had been completed or started, the National Climate Change Team/Committee included consideration of the infectious disease health risks of climate change (Figure 4B). The accuracy of this particular response was verified through a review of all fifth submitted National Communications to the UNFCCC provided by each member state from the end of 2009 to the end of 2010 (UNFCCC 2010). A 100% concordance was found between the statements of the experts regarding the health contents of the UNFCC communications and the actual contents of these reports. National preparedness measures reported by the government officials are listed in Table 3, and an assessment of the effectiveness of institutions that monitor and provide health services for infectious diseases is provided in Figure 5. Seventeen countries reported plans to respond to the potential threats from climate change–sensitive infectious diseases through surveillance, monitoring, regulations, resource allocation, or communication strategies.

**Table 3:** Planning and preparedness for infectious disease threats attributed to climate change in Europe, according to infectious disease experts, 2009/2010.

Questions on planning and preparedness for infectious disease threats	Yes/total (%)
Has your country completed a national assessment of the potential health impacts of climate change?		9/27 (33)
If no (*n* = 18):		
Is your country planning or currently conducting a national assessment?		3/12 (25)
Are there regional/local planning and coordination institutions to monitor and control climate-sensitive infectious diseases?		14/29 (48)
Does your department have plans over the next 5 years for research on and response to climate-sensitive infectious diseases?		17/29 (58)
If yes (*n* = 17):		
Are there plans to alter current vector-borne disease surveillance and control programs to address the threats of climate change? This includes changing the frequency or location of monitoring and surveillance programs to detect changes in geographic range or incidence.		15/17 (88)
Are there plans to alter monitoring of water sources or water treatment regulations to address the threats of climate change?		4/16 (25)
Are there plans to alter food safety and other regulations to address the threats of climate change?		3/14 (21)
Are there plans to increase the human and material resources devoted to climate change risks?		6/17 (35)
Does your National Climate Change Team/Committee explicitly include consideration of the infectious disease health risks of climate change?		14/25 (56)
Did you or your department participate in the last two meetings of your National Climate Change Team/Committee?		8/16 (50)
Does the National Climate Change Team/Committee have a strategy for communicating the risks of climate change to the geographic range and incidence of climate-sensitive infectious diseases?		5/13 (39)

*Surveillance activities.* Several member states reported national, regional, or local surveillance activities (e.g., sentinel or cluster surveillance) for diseases they consider to be climate sensitive in addition to routine surveillance for EU-reportable communicable diseases (European Commission 1999). For example, five countries (Czech Republic, France, Hungary, Latvia, and Norway) reported surveillance of TBE, one of them since 1953; four countries (Czech Republic, France, Latvia, and Norway) reported surveillance of Lyme borreliosis at least on a regional level; and three countries reported surveillance of hantavirus infections (France, Hungary, and Latvia). Other pathogens covered by nonmandatory surveillance activities in at least one country included Crimean-Congo hemorrhagic fever, dengue fever, echinococcosis, human granulocytic anaplasmosis, hemorrhagic fever with renal syndrome, Mediterranean spotted fever, *Naegleria fowleri* infections, norovirus infections, Rift Valley fever, Q fever, rotavirus infections, and yellow fever.

Government officials also assessed current surveillance programs, diagnostic support, collaboration with the veterinary sector, and outbreak response (Figures 4D and 5). A number of respondents reported the need for at least some improvement in these programs, particularly in current surveillance systems (83%) and collaboration with veterinary services (69%).

## Discussion

In the absence of scientific certainty, expert assessment pools informed opinion and can add support for findings it corroborates while reducing support for findings it does not. Thus, expert judgment can help policy makers prioritize areas for action and make informed decisions (Weir et al. 2010). This is especially important for the epidemiology of climate change, for which large methodological challenges and research gaps exist (Xun et al. 2010). However, confidence in expert opinion hinges on the validation of the experts’ specialized knowledge; here, we assess the reliability of some of the expert opinions in relation to the peer-reviewed literature.

*Vector-borne diseases.* The experts surveyed generally noted an expansion in the range of infectious diseases. In particular, respondents from northern countries, compared with those from southern countries, considered the edge of the geographic distribution limits of vector-borne diseases, such as Lyme borreliosis and TBE, to be susceptible to the effects of climate change (Figure 1A,B). This appraisal is consistent with findings from the peer-reviewed literature; for example, the main European tick vector, *Ixodes ricinus*, lives for several years and has been observed to have markedly changed its latitude distribution (northern Sweden) and altitude distribution (the Czech mountains) over the last 30 years (Daniel et al. 2009; Talleklint and Jaenson 1998). These distribution changes have been reported to be correlated with changes in the length of seasons (Jaenson and Lindgren 2011), climatic variations (Materna et al. 2005), and the number of degree-days in different seasons with temperatures of importance for the activity and survival of the vector (Lindgren et al. 2000). The experts who participated in the survey stated that with climate change, Lyme borreliosis is expected to change its altitude and latitude distribution (Figure 1A). This is in accordance with the literature regarding both observed relationships between climate variations and tick-borne disease incidence over the last four to five decades (Daniel et al. 2009; Kaiser 1995; Lindgren and Gustafson 2001) and projected changes (Jaenson and Lindgren 2011), which estimate Lyme borreliosis becoming more prevalent in northern and central Europe and expanding to higher latitudes and altitudes. However, contributing factors other than climate, such as socioeconomic changes, may play important roles as well, particularly in central and eastern regions of Europe (Randolph 2008).

Emerging concerns for some of the vector-borne diseases, in particular chikungunya and dengue fevers, were reflected in the differences in answers between the survey periods (Table 1). This could be explained in part by increased awareness among experts in the wake of recent outbreaks, such as the first-ever European chikungunya outbreak in Italy in 2007 (Rezza et al. 2007).

Experts reported observed changes in their countries in the seasonality of hantavirus (Figure 1C). In the Nordic countries, larger outbreaks of hantavirus have been associated with lack of snow cover, which forces rodents, the vector of hantavirus, to move closer to and inside human buildings. Large epidemics occurred, for example, during the exceptionally mild and snowless winter of 2006/2007 in northern Sweden (Evander and Ahlm 2009).

Leishmaniasis transmitted by sandflies is currently prevalent only in southern Europe, where the experts did not consider it likely to be affected by climate change (Figure 1D). Sandfly vectors, as well as the protozoa, are very sensitive to ambient temperatures. In fact, there are indications that the geographic distribution of leishmaniasis has expanded, and locally infected cases have now been documented in southern Germany (Lindgren et al. 2006; Naucke and Schmitt 2004).

*Food- and water-borne diseases.* Water-borne diseases display a strong seasonality (Schijven and de Roda Husman 2005), and experts in northern and eastern Europe considered these diseases likely to be affected by climate change (Figure 3A,B). Leptospirosis can be transmitted by water contaminated with urine and fecal matter from infected animals, such as the floodwaters in the Czech Republic in 1997.

Projected increases in the intensity and frequency of rainfall in the northern regions could lead to *Cryptosporidium* infiltration in water-treatment and distribution systems (Semenza and Nichols 2007). Respondents from northern European countries reported a potential increase in climate change risk, as opposed to those from southern European countries, where projected decreases in precipitation could reduce these risks (Figure 3B). However, these observations also reflect reporting bias; those countries with better EU *Cryptosporidium* notifications reported a climate change risk, whereas those countries with incomplete (or no) *Cryptosporidium* notifications considered the risk to be low (Semenza and Nichols 2007).

In this expert survey, outbreaks of water-borne diseases were considered less likely to be affected by climate change than food- or vector-borne diseases. A total of 23 countries reported one or more water-borne disease outbreaks due to drinking water contamination in the last decade, with 12 reporting five or more outbreaks. For most countries, these numbers amounted to less than one outbreak every few years. No respondents thought that past water-borne disease outbreaks due to contaminated drinking water were attributable to actual climate change; however, extreme weather events due to climate variability could be implicated. Low numbers of outbreaks of water-borne diseases are a testament to high-quality water treatment in Europe, but this might also be due to underreporting because diarrheal symptoms are reported to health care providers only if they are severe.

Food-borne infections linked with climate change were reported largely from central and eastern Europe. *Salmonella* and *Campylobacter* incidence display a distinct seasonal pattern that has been associated with climate variability (increased temperatures, heat waves, and flooding) (Bentham and Langford 2001; Kovats et al. 2004, 2005; Lake et al. 2009). However, animal control measures and other public health interventions have led to decreasing risk in several European countries, which may overshadow any potential effects of climate change. Survey respondents did not attribute food-borne outbreaks in their countries to climate change, despite evidence of strong correlations between temperature and disease incidence reported in the literature (Kovats et al. 2004).

*Planning and preparedness.* Regional climate change impacts are a function of local vulnerabilities, exposure, and changing weather variables. The ability to adapt depends on a number of factors, such as surveillance information, human resources, available technology, institutional capacity, economic resources, social equity, and political will (Huang et al. 2011). In our analysis we attempted to capture some of these planning and preparedness activities. The majority of experts (17 of 29) acknowledged research on climate-sensitive infectious diseases in their countries; however, few institutions are in place to monitor climate-sensitive infectious diseases (14 of 29), and the majority of these institutions need improvement. Respondents from only seven countries indicated that their country had conducted a national climate change assessment that covered infectious diseases extensively or adequately, but one-third of the countries with a response plan were anticipating the need for increasing human and material resources to address risks from climate change (Figure 4 and Table 3).

*Surveillance activities.* Approximately 80% of government officials indicated that their current surveillance activities needed at least some improvement (Figure 4D). Establishing syndromic surveillance systems (which monitor health-related data that precede diagnosis, such as emergency calls, school absenteeism, pharmacy-based drug sales, and Internet queries) could be one way of enhancing surveillance because they can capture real-time trends, geographic spread, or outbreaks that would otherwise go unnoticed, thereby complementing ongoing surveillance activities. Surveillance of potential new risk regions where climate-sensitive pathogens or disease vectors may become introduced and established is also of considerable importance, such as for dengue fever surveillance in the Mediterranean region. Vector, environmental, or drinking water surveillance are other approaches that could be considered.

*Limitations.* Our study has a number of limitations. Expert opinions can be vulnerable to recall bias, susceptible to institutional or disciplinary biases, shaped by recent disease outbreaks, influenced by increased media reporting, and so on. Experts in this study were officially nominated by government agencies as the point person for climate change in their country (ECDC 2011), as is standard practice for other infectious disease programs within the ECDC. However, no independent assessment of their professional or academic expertise was conducted. The data collected were not subjected to third-party verification and might thus contain inaccuracies and misconstructions. Nevertheless, we compared the different survey results with data from the peer-reviewed literature and the national reports sent to the UNFCCC (2010). The survey result was remarkably consistent with the other sources we examined, with the exception of food-borne diseases, which highlights potential food security issues (food safety, food production, etc.) related to climate change. This expert assessment of national representatives likely corresponds with the government opinion on this topic. Although the data represent perceived impacts, our expert analysis can also shed light on the extent of scientific consensus and thus inform public policy (Van Rij 2010).

## Conclusion

Attributing single infectious disease epidemics to climate change is not possible, but longer-term trends in disease outbreaks and incidence may signal linkages to climate variations. The exact attribution of changes in specific infectious disease risks to climate change is probably not attainable. Nonetheless, public health practitioners are obliged to address credible risks—even if that requires acting in the absence of conclusive evidence.

Expert opinion can provide pivotal insights and guide climate change adaptation in a field with complex interacting drivers (Suk and Semenza 2011). National expert opinion concerning risks of vector- and water-borne diseases from climate change matched well with data from the peer-reviewed literature, but less well for food-borne diseases where the causal link has a range of potential confounders. National climate change assessments were reported mainly from Western Europe, and a number of institutional weaknesses were identified, such as research on and control of climate-sensitive infectious diseases. Most noticeable, however, was the need to improve current surveillance of infectious diseases. Current deficiencies are of particular concern given budgetary shortfalls for infectious disease programs during economic crises, as we documented recently (Rechel et al. 2011; Suhrcke et al. 2011). The ECDC developed a handbook for climate change impact, vulnerability, and adaptation assessment to assist member states with this process (ECDC 2010b). Ultimately, vigilant surveillance, the cornerstone of public health practice, will likely be ever more important for addressing climate change threats.
